# Apparatus for Neutron Scattering Measurements on Sheared Fluids

**DOI:** 10.6028/jres.094.024

**Published:** 1989

**Authors:** G. C. Straty

**Affiliations:** National Institute of Standards and Technology, Boulder, CO 80303

**Keywords:** fluids, neutron scattering, rheology, SANS, shear

## Abstract

We report on the construction of an apparatus to allow neutron scattering measurements on fluids undergoing shear. The apparatus has been used with the cold neutron small-angle-neutron-scattering (SANS) spectrometer at the NIST research reactor and will be made available to users as a permanent part of the NIST facility.

## 1. Introduction

It is now well established that investigations of fluids subjected to a shear are rewarding. For example, studies of the structural changes induced by shearing motions lead to a better understanding of long-range intermolecular correlations in liquids [1], and allow the investigation of the alignment and intramolecular changes of condensed phase polyatomic molecules [2], and the phase changes and thermodynamic properties of liquids out of equilibrium [3]. The relationship of theory, especially nonequilibrium computer simulation, with experiment is particularly rich in this area and the two complementary approaches have given fresh insight into the origin of non-Newtonian behavior in liquids and the basis of rheology and fluid dynamics [4].

This note is to report on the construction of an apparatus which allows neutron scattering measurements to be made on fluids undergoing shear [5]. The apparatus has been used with the small-angle-neutron-scattering (SANS) spectrometer at the NIST research reactor and will be available to users on a permanent basis. Full details on the apparatus and the results of preliminary experiments will be presented in a future publication.

## 2. Apparatus

**Figure 1:**
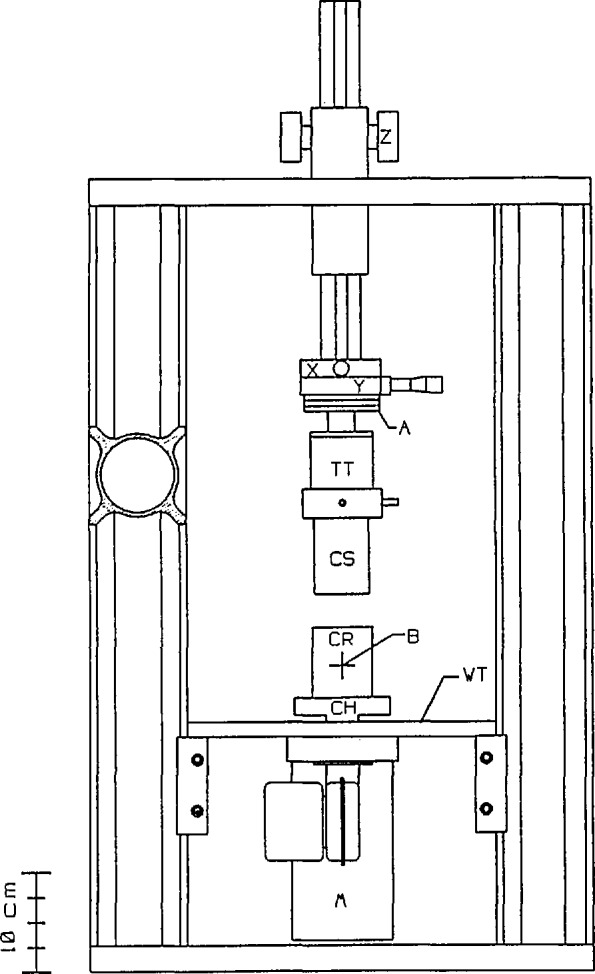
Schematic drawing of the shearing apparatus: M—Drive Motor, CR—Cell Rotor, CS—Cell Stator, TT—Torque Transducer, A—Alignment Coupling, X—Micrometer Adjust, Y—Micrometer Adjust, Z—Vertical Adjust, WT—Work Table, CH—Cell Chuck, B—Beam Center. See text for details.

The apparatus consists of a Couette-type concentric cylinder sample cell coupled to a computer automated drive and thermal control system [6]. Neutrons are scattered from the fluid contained within the annular gap between the cylinders. The apparatus is shown schematically in figure 1.

The rigid aluminum frame consists of vertical rails attached to heavy plates at bottom and top. The vertical rails are commercial structural elements of the type commonly used for optical bench construction. They have a unique cross section which allows the attachment of numerous commercially available slides, carriages, clamps, etc. allowing for easy attachment of components and accessories. A work table (WT) is attached to the rails and supports the drive motor (M). The table is drilled and tapped in a regular pattern to provide an easy method for mounting of accessories. The cell chuck (CH), mounted on the motor shaft, can accommodate cells of various sizes and has provision for precise alignment of the cell rotor (CR) axis and the motor axis. Vertical adjustment of the cell stator (CS) is accomplished with a commercial rack-and-pinion assembly (Z), which was extensively modified to improve rigidity and stability. Parallelism between the motor and stator axes is obtained by adjustment of an aligning coupling (A). Collinearity is achieved using a pair of micrometer actuated translation stages (X) and (Y). An optional torque transducer (TT) can be used to obtain rheological information about the samples.

**Figure 2:**
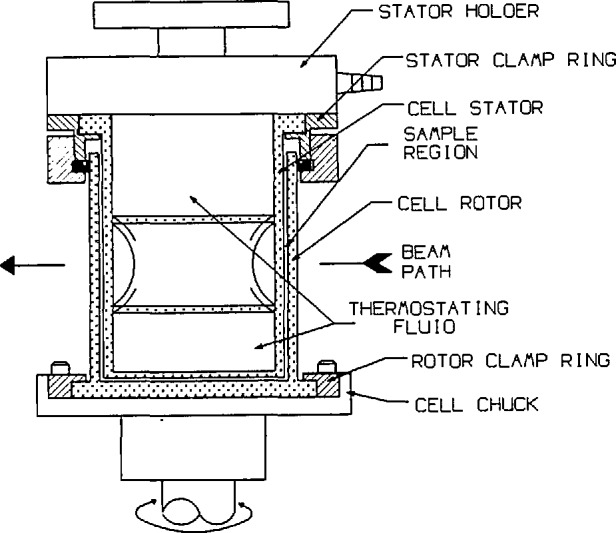
Detailed drawing of the shearing cell.

A more detailed schematic of the cell is shown in figure 2. The cell consists of a stationary cylindrical quartz glass stator surrounded by a rotating cylindrical quartz glass rotor. Quartz exhibits low and uniform scattering at small angles and is ideal for SANS experiments. The cell used for our preliminary work has a mean diameter of about 56.5 mm and an annular gap of 0.5 mm for the samples.

Thermostating fluid is circulated within the stator for temperature control. Temperatures are measured using a platinum resistance thermometer in contact with the thermostating fluid in the stator. The thermostating fluid is excluded from the neutron beam path by the cross-tube located inside the stator. The nominal operating temperature range for the apparatus is currently 245–373 K and is determined primarily by the range of the thermostating fluid bath controller. At elevated temperatures the motor can be cooled using an optional cooling fan.

The drive motor is a state-of-the-art dc brushless servo motor. These motors have permanent magnet armatures and the fields are commutated by computer controlled switching using position feedback from a shaft resolver integral with the motor shaft. This technology allows smooth, continuous rotations under programmed conditions of acceleration, velocity, torque (velocity limited), direction, position, angle, start, stop, delay, time and any sequence thereof. These capabilities allow the precise control of all rotational parameters to an accuracy of better than 0.1% over extended periods of time.

The controlling computer can be any of a number of PCs available today. Computer hardware and software are available to allow completely automatic operation of the shearing apparatus in parallel with the existing on-line data acquisition system at the NIST reactor.

**Figure 3:**
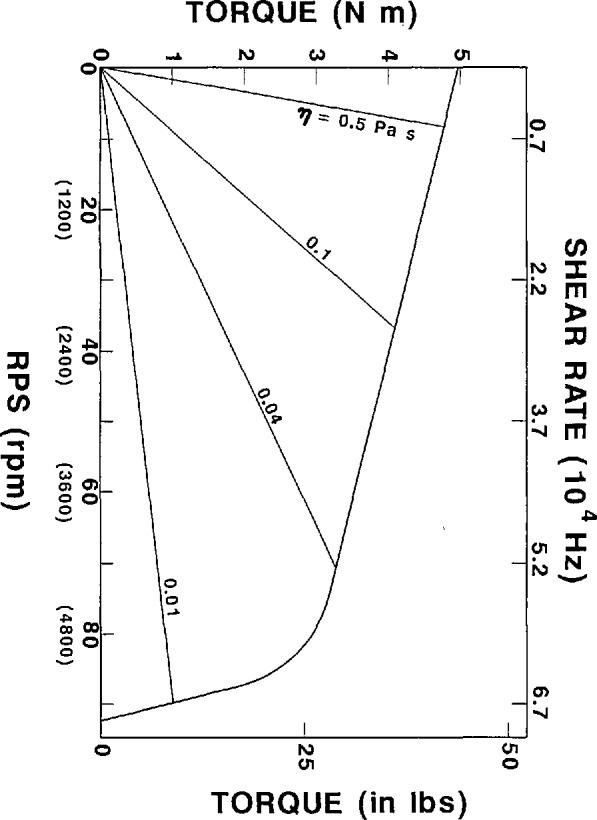
Plot of the torque as a function of the rotation speed and of viscosity for various viscosities. Viscosity and shear rate data are dependent on geometry and are calculated for the cell as described in the text.

A velocity-torque curve for the drive system is shown in figure 3. The viscosity and shear rate data are geometry dependent and are calculated for the cell dimensions as described. The torques at high RPMs are upper bounds based on the motor specifications and may be limited in an actual experiment because of hydrodynamic constraints peculiar to any given sample and the additional load imposed by the dynamics of the rotating elements. The apparatus is designed to accommodate other cell geometries to allow more versatility in the applicable range of shear rates and torques.
